# Running in highly cushioned shoes increases leg stiffness and amplifies impact loading

**DOI:** 10.1038/s41598-018-35980-6

**Published:** 2018-11-30

**Authors:** Juha-Pekka Kulmala, Jukka Kosonen, Jussi Nurminen, Janne Avela

**Affiliations:** 1 Motion Analysis Laboratory, Children’s Hospital, University of Helsinki and Helsinki University Hospital, Helsinki, Finland; 2 John A. Paulson School of Engineering and Applied Sciences, Harvard University, Cambridge, MA USA; 3 Neuromuscular Research Center, Biology and Physical Activity, Faculty of Sport and Health Sciences, University of Jyväskylä, Jyväskylä, Finland

## Abstract

Running shoe cushioning has become a standard method for managing impact loading and consequent injuries due to running. However, despite decades of shoe technology developments and the fact that shoes have become increasingly cushioned, aimed to ease the impact on runners’ legs, running injuries have not decreased. To better understand the shoe cushioning paradox, we examined impact loading and the spring-like mechanics of running in a conventional control running shoe and a highly cushioned maximalist shoe at two training speeds, 10 and 14.5 km/h. We found that highly cushioned maximalist shoes alter spring-like running mechanics and amplify rather than attenuate impact loading. This surprising outcome was more pronounced at fast running speed (14.5 km/h), where ground reaction force impact peak and loading rate were 10.7% and 12.3% greater, respectively, in the maximalist shoe compared to the conventional shoe, whereas only a slightly higher impact peak (6.4%) was found at the 10 km/h speed with the maximalist shoe. We attribute the greater impact loading with the maximalist shoes to stiffer leg during landing compared to that of running with the conventional shoes. These discoveries may explain why shoes with more cushioning do not protect against impact-related running injuries.

## Introduction

Running, a popular exercise across the world, offers significant cardiovascular and other health benefits^1^. However, each year between 37% and 56% of runners worldwide incur injuries^2^ that typically result from repeated loading of the musculoskeletal system. In particular, when the foot hits the ground, the magnitude of the vertical ground reaction force impact peak (IP) and loading rate (LR) have been linked to the risk of running injuries^3,4^, so the study of running injury prevention has primarily focused on the management of impact loading.

In order to reduce the risk of running-related injuries, running shoe manufactures have added cushioning to shoe soles aimed at reducing impact loading. However, studies show no evidence of reduced running injury rates with increasing amounts of cushioning^5–8^. The explanation for this counterintuitive finding may lie in the well-recognized, but poorly understood phenomenon that highly cushioned shoes have a limited ability to reduce impact loading^6,9^. In fact, some studies have noted even a slight increase in impact loading when running in shoes with a compliant versus a hard midsole^10–13^. These findings counters the impact attenuation theory^14^ and the results of *in vitro* mechanical impact tests^15^, both of which indicate a significant reduction in impact loading with increased cushioning. Our goal in this paper is to shed new light on understanding the mechanisms that might be responsible for countering the impact attenuation effect of extra shoe cushioning during running.

**Figure 1:**
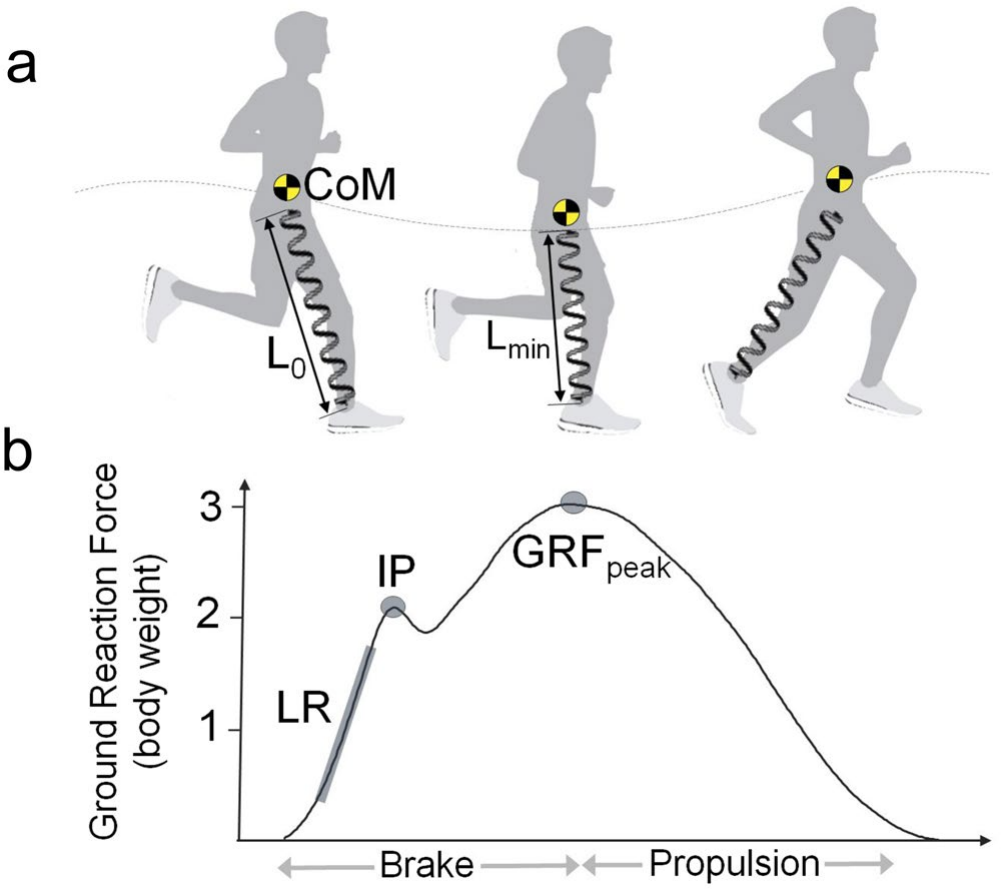
Spring-mass mechanics of running. (**a)** The mechanical energy during the braking phase of running is absorbed by compression of the leg-spring from initial length (L_o_) to minimal length (_Lmin_). The body’s centre of mass (CoM) reaches its highest position during the aerial phase, whereas the lowest position, L_min_ and the (**b**) peak ground reaction force (GRF_peak_) occur at the mid-stance. Leg stiffness can be calculated as a ratio of GRF_peak_ to the change in leg length. During heel running, a visible GRF impact peak (IP) and a relatively high impact loading rate (LR) occur after the heel collides with the ground.

Previous research^16^ has identified elastic leg behaviour as critical to terrestrial locomotion. During running, the leg undergoes compression in the first half of the stance while gradually decelerating the body and then recoils in the second half of the stance to reaccelerate the body. This cyclic behaviour permits efficient force production through a stretch-shortening muscle action^17^ and is essential for avoiding mechanically costly high-energy impacts during foot–ground contact^18^. The elastic leg behaviour during running can be described as a simple spring-mass system, where a leg-spring supports the point mass representing the runner’s centre of mass (CoM) (Fig. 1)^19–21^.

Importantly, studies using the spring-mass model have shown that running humans maintain the same bouncing movement of the body’s CoM across surfaces with different stiffnesses by adjusting their leg stiffness during the stance phase^22,23^. For example, when transitioning from a hard surface to a more compliant surface, a runner’s leg become stiffer and compresses less to maintain the preferred spring-mass mechanics. Notably, few other studies in human running have established that, similar to shoes with different amounts of cushioning, surfaces with different stiffness properties have a limited effect on the ground reaction force IP and LR^24,25^. Because the ground reaction force reflects the acceleration of the entire body mass (F = ma), researchers have inferred^24,25^ from data that the maintenance of nearly constant IP and LR, despite having different impact interfaces, must somehow result from the running mechanics adjustments that cancel out the impact attenuation effect of a compliant surface. However, the underlying mechanisms of this phenomenon are not fully understood.

Previous observations led us to hypothesize that the runner’s leg may stiffen and compresses less when running in shoes with additional cushion, which in turn, might be responsible for countering the impact attenuation effect of the extra cushioning. To test this, we examined ground reaction forces and the spring-like mechanics of running in shoes with conventional (CON) and maximalist (MAX) cushioning at two training speeds, 10.0 km/h and 14.5 km/h.

## Results

### Highly cushioned shoes increase impact loading during running

We recruited 12 healthy men (mean age 27) who had several years of sports background and who ran with a heel strike pattern. For a MAX shoe in this study, we used the Hoka Conquest running shoe (Hoka One One, Marina Bay, CA, USA) and as s CON shoe we used the Brooks Ghost 6 running shoe (Brooks Sports, Inc., Seattle, WA, USA). After a warm-up, each participant underwent a 3D running analysis with CON and MAX shoes at the slower (10 km/h) and faster (14.5 km/h) speeds (see Methods section for details). The participants demonstrated no differences in step length, contact time or cadence between CON versus MAX conditions (Table 1).

**Table 1:** Mean (SD) data for the CON and MAX shoes at slow and fast running speeds.

	Slow speed (10 km/h)					Fast speed (14.5 km/h)				
	CON shoe		MAX shoe		*t*-test	CON shoe		MAX shoe		*t*-test
*Step parameters*										
Running speed (km/h)	10.2	(0.3)	10.1	(0.3)	0.431	14.6	(0.3)	14.5	(0.3)	0.423
Step length (m)	1.08	(0.06)	1.06	(0.07)	0.202	1.43	(0.08)	1.42	(0.10)	0.438
Contact time (ms)	259	(22)	264	(25)	0.194	217	(22)	215	(25)	0.563
Cadence (step/min)	160	(9)	161	(8)	0.453	166	(9)	167	(11)	0.601
*Spring-like mechanics*										
Leg stiffness (kN/m^−1^)^++^	23.3	(6.2)	23.9	(6.7)	0.189	25.5	(7.0)	27.0	(7.7)	0.009**
Leg compression (mm)^++^	82.8	(11.6)	79.5	(12.2)	0.030*	82.6	(12.8)	80.1	(13.9)	0.006**
Body’s CoM descent during stance (mm)^++^	−60.6	(7.4)	−58.5	(6.4)	0.006**	−56.4	(7.3)	−54.0	(6.7)	0.030*
Body’s CoM total oscillation (mm)^#^	87.4	(12.2)	84.1	(11.2)	0.082	89.6	(14.4)	92.3	(15.7)	0.120
Peak vertical GRF (BW)^##^	2.60	(0.20)	2.55	(0.26)	0.067	2.85	(0.26)	2.91	(0.26)	0.035*
*Vertical GRF impact loading*										
Impact peak (BW)^#,+++^	1.60	(0.17)	1.71	(0.19)	0.001***	2.01	(0.32)	2.25	(0.32)	0.001***
Average loading rate (BW/s)^+^	42.0	(13.0)	44.9	(10.0)	0.214	59.0	(15.2)	67.3	(14.6)	0.038*

Univariate difference between shoe conditions (*t*-test): *p < 0.05 and **p < 0.01.

Shoe by speed interaction effects (a two-way repeated measures ANOVA): ^#^*p* < 0.05, ^##^*p* < 0.01 and ^###^*p* < 0.001.

The main effect for shoe conditions (a two-way repeated measures ANOVA): ^+^*p* < 0.05, ^++^*p* < 0.01 and ^+++^*p* < 0.001.

CoM = Centre of Mass, BW = Body weight^,^ GRF = ground reaction force.

**Figure 2:**
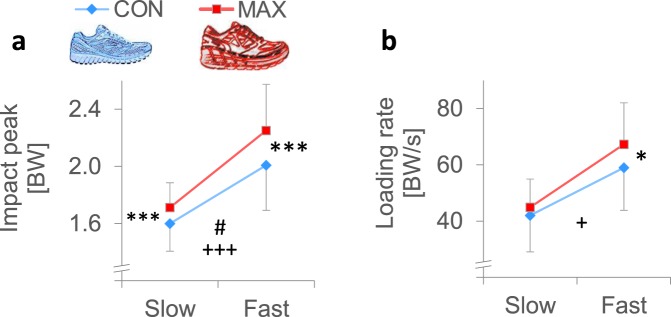
Mean data (SD) of the vertical ground reaction force (**a**) impact peak (IP) and (**b**) loading rate (LR) for the CON and MAX shoes at slow (10 km/h) and fast (14.5 km/h) running speeds. Univariate difference between shoe conditions (*t*-test): *p < 0.05 and ***p < 0.001. Shoe by speed interaction effects (two-way repeated measures ANOVA): ^#^*p* < 0.05. The main effect for shoe conditions (two-way repeated measures ANOVA): ^+^*p* < 0.05 and ^+++^*p* < 0.001.

Heel running causes a visible IP and a relatively high LR. We examined these two measures to compare the magnitudes of impact loading between running with a CON and MAX shoes. We found significant main effects for impact LR (*p* = 0.049) and IP (*p* = 0.001) rate; both showed greater values of loading when running with MAX shoes versus CON shoes (Table 1, Fig. 2a,b).

A closer analysis revealed that the difference between shoes in the impact load parameters were more apparent at faster than slower running speeds. At the slower running speed, only the IP was significantly different between shoes (6.4% more IP in the MAX shoes; *p* = 0.001), whereas at faster speeds, the MAX shoe demonstrated IP (*p* = 0.001) and LR (*p* = 0.038) values that were 10.7% and 12.3% greater, respectively, than those with the CON shoe (Fig. 2a,b).

### Highly cushioned shoes change the spring-like mechanics of running

**Figure 3:**
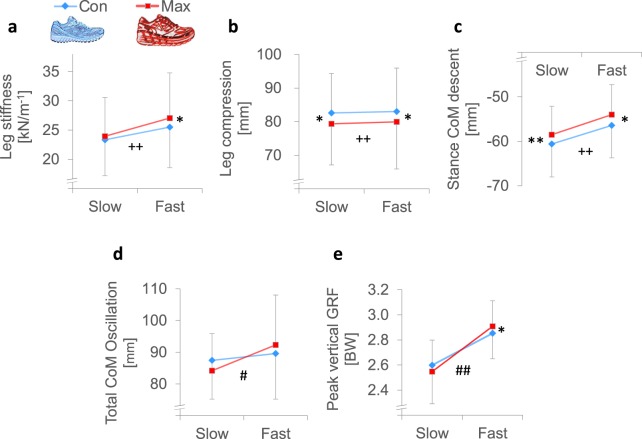
Mean data (SD) of the (**a**) leg stiffness, (**b**) leg compression, (**c**) CoM decent during stance, (**d**) total CoM oscillation and (**e**) peak vertical GRF for the CON and MAX shoes at the slow (10 km/h) and fast (14.5 km/h) running speeds. Univariate difference between shoe conditions (*t*-test): *p < 0.05 and **p < 0.01. Shoe by speed interaction effects (a two-way repeated-measure ANOVA): ^#^*p* < 0.05 and ^##^*p* < 0.01. The main effect for shoe conditions (a two-way repeated measures ANOVA): ^++^*p* < 0.01.

Using a spring-mass model^26^, we determined the stiffness of the runner’s leg during the ground contact when wearing MAX shoes and CON shoes. The results revealed a significant main effect (*p* = 0.007) for shoe conditions, showing greater leg stiffness for the subjects running with MAX shoes (Fig. 3a, Table 1). This extends the findings from earlier work^22,23^ by showing that leg stiffness during running can change not only due to surface stiffness, but due to the level of shoe cushioning. The analysis of slow and fast running speeds revealed that the increase in the leg stiffness in the MAX shoe was more pronounced at faster (*p* = 0.009) than slower (*p* = 0.189) running speeds (Fig. 3a).

Because leg stiffness is a product of ground reaction force and leg compression, we examined these two measures separately to address the mechanisms responsible for the differences in leg stiffness observed in shoe conditions. We found that runners wearing MAX shoes compressed their legs significantly less across slow (3.2 mm, *p* = 0.030) and fast (3.1 mm, *p* = 0.006) running speeds than they did when wearing CON shoes (Fig. 3b). By contrast, we found that runners using the MAX shoes showed a speed-dependent response in the peak vertical ground reaction force (shoe by speed interaction *p* = 0.009): at the slow speed in MAX shoes, runners applied 38 N less force on the ground than in CON shoes, whereas at the fast speed, the force was 41 N greater in MAX shoes (Fig. 3e). This indicates that the more pronounced increase in leg stiffness observed with MAX shoes at faster running speeds was due to differences in the ground reaction force rather than changes in leg compression.

From the 3D motion analysis data, we then determined the body’s CoM vertical movement for running. Due to a thicker midsole in MAX versus CON shoes, the height of the body’s CoM was slightly higher throughout the stride cycle when running in MAX shoes (Supplementary Fig. S1). During stance, the maximal decent of runner’s CoM was consistently lower with MAX shoes at slow (2.1 mm, *p* = 0.006) and fast (2.4 mm, *p* = 0.030) running speeds compared to CON shoes (Fig. 3c), which corresponds to the observed reduction in leg compression when running with MAX shoes. However, the total body CoM vertical oscillation, calculated from the lowest point of the ground contact to the highest point of the flight phase, showed a speed-dependent response to additional shoe cushioning (shoe by speed interaction *p* = 0.045). Although we observed a trend toward a lower total displacement (3.3 mm less in MAX shoes, *p* = 0.082) at the slow running speed, we observed the opposite at the faster running speed (2.8 mm more in MAX shoes, *p* = 0.120) (Fig. 3d). These data highlight that running speed can significantly influence the way runners adapt to different shoe-cushioning conditions.

## Discussion

Our results demonstrate that running in highly cushioned MAX shoes amplifies rather than attenuates impact loading, as both the IP and LR increased relative to that in the CON shoes, at a running speed of 14.5 km/h. However, the MAX shoes had smaller effects on impact loading at the slower speed (10 km/h), since only the IP slightly increased compared to running with CON shoes. Our finding of greater impact loading when running in shoes with maximalist cushion conflicts with common assumptions^27^ that additional cushioning should decrease impact loads. Several studies^10–13^ have also noted a slight increase in IP and LR for runners in shoes with a compliant versus a hard midsole, but a mechanistic explanation for this phenomenon has remained poorly understood.

A possible reason for the increased IP and LR for MAX shoes may be the fact that we observed significant differences in the spring-like running mechanics between the MAX and CON shoes. Notably, we found that when wearing MAX shoes, the runners’ legs became stiffer due to lower compression compared to runners wearing CON shoes. The stiffer leg during the landing phase in MAX shoes can diminish the impact attenuation effect gained from the additional cushioning by decelerating the body’s entire mass more rapidly as compared to more compliant landing with CON shoes. Adjustments in leg stiffness when wearing MAX shoes align with those seen when runners transition from a hard surface to a more compliant surface^22,23^, suggesting a similar adaptation mechanism to maintain the preferred body CoM support mechanics.

Running with MAX shoes also altered the peak vertical force and body’s CoM oscillation relative to running with CON shoes, and unexpectedly, these changes differed at slow and fast running speeds. Thus, although the preferred support mechanics principle^22^ provides a logical explanation for the observed leg adjustments in the stance phase in MAX shoes, it does not explain why the dynamics of bounce, reflected in peak vertical force and CoM oscillation, was different for runners in MAX shoes at slow versus fast running speeds. Nevertheless, it is reasonable to believe that speed-dependent adaptation to a MAX shoe reflects runners’ tendency to adjust the dynamics of bouncing gait to the shoes’ mechanical characteristics. Although not measured in the present study, higher midsole compliance of the MAX shoe likely resulted in lower natural frequency (i.e. longer midsole compression-recoil time) compared with the CON shoe. This together with the fact that the force-time characteristics of the running gait differ significantly at slow and fast speeds may potentially explain speed-dependent adaptation to a MAX shoe. At the slow running speed, there is a relatively long contact time (i.e. ~0.26 s) available for the runner to apply force on the ground, but at the fast running speed, the runner must apply greater force (~0.3 BW) on the ground over a much shorter time period (i.e., ~0.22 s). The slightly smaller CoM oscillation and consequently lower force application of runners wearing a MAX shoe at slow speeds may be an innate tuning mechanism to restrain the shoe’s midsole compression, which allows the runner to match the shoe midsole compression-recoil timing to that of his spring-like legs. Conversely, the opposite — a slightly increased force application and CoM oscillation — is observed at the faster running speed with MAX shoes, which may reflect a mechanism to limit contact time by speeding up midsole compression-recoil behaviour, enabling a quick transition from braking phase to the propulsion phase. For confirming these theories, we call for further research examining shoe-runner interaction across different running speeds and identify several future directions that can build on our findings.

First, because mechanical shoe characteristics were not measured in this study, it remained unclear how the actual shoe cushion properties differed between NORM and MAX shoes. Therefore, the shoe mechanical testing together with running mechanics data are needed to further elaborate shoe-runner interaction. Second, the body’s CoM vertical displacement was estimated based on the 3D movement data rather than the force plate method^28^. Although, previous literature^29,30^ indicate a very good agreement in the CoM movement obtained using the 3D approach and the force plate method, no studies have compared the results of these two techniques when running in shoes with different cushioning properties. Finally, of note is that although our leg stiffness values are well in line with other studies^26,30,31^ using the direct 3D method, they are generally 25–55% greater than those^21–23,32^ calculated with a traditional spring-mass model^19,20^. This discrepancy arises primarily from a much lesser leg compression values in studies using the direct 3D method (~7–9 cm) versus those using a traditional spring-mass model (~12–14 cm) rather than differences in the GRF. We believe that the direct 3D method may provide a more accurate representation of the true leg compression, because it avoids assumptions made by a planar spring-mass model^19,20^ that the leg compression can be estimated from running speed, ground contact time, and leg length at initial contact.

The observed running mechanics adjustments in the present study resolve the shoe cushioning paradox and also point towards importance of speed-specific optimization of the shoe properties in order to improve running injury prevention. Our findings that MAX shoes amplify rather than attenuate impact loads particularly at the faster (14.5 km/h) running speed suggest an increased risk of impact-related injuries compared to CON running shoes at the same speed. On the other hand, we found that MAX shoes little affected impact loading at the slower running speed (10 km/h), which suggests a minor effect on the risk of injuries. Notably, studies examining the relationship between shoe cushioning and injuries^8^ and impact load magnitude and injuries^4^ have focused on leisure distance runners, who rarely run at speeds greater than 10 km/h. For example, Theisen *et al*.^8^ found similar injury rates in runners who used shoes with soft versus hard midsoles, but the reported mean running speed in both shoe groups was ~9.5 km/h. As a result, a soft versus hard midsole shoes in their study likely had a minimal effect on the impact loading, which can explain similar injury rates between the two shoe groups. Consequently, running speed should be considered in future studies that examine whether the type of shoe cushioning influences running injuries.

## Methods

### Participants

Twelve healthy male subjects (age, 27 ± 5 years; height, 179 ± 4 cm; weight, 75 ± 6 kg, leg length 82 ± 3 cm) were recruited for this study. Each subject had sports experience (team sports, running), several years of training and ran with a heel striking pattern. The subjects provided informed consent and confirmed that they did not have a recent history of musculoskeletal problems, such as a recent injury or surgery that could affect their running patterns. This study was approved by the ethics committee of University of Jyväskylä, and performed in accordance with the Declaration of Helsinki.

### Experimental setup

For the highly cushioned shoe in this study, we used the Hoka Conquest men’s running shoe (Hoka One One, Marina Bay, CA, USA), as the maximalist (MAX) shoe. This shoe had a 43 mm heel and 37 mm forefoot height, respectively, (heel-toe drop of 6 mm), and its measured weight was 321 g. The Brooks Ghost 6 men’s running shoe (Brooks Sports, Inc., Seattle, WA, USA) was the conventional (CON) cushioned running shoe used in this study. This shoe weighs 301 g, and has a 33 mm heel and 22 mm forefoot height, respectively (heel-toe drop of 12 mm).

After a 400 m warm-up period with their own shoe, each the subjects performed test trials using MAX and CON shoes. The shoe order was randomised, and the subjects performed at least three practice running trials along a 30 m track at each speed to familiarize themselves with the shoes prior to data collection. Three valid trials were then recorded first at 10.0 (±0.3) km/h and then at 14.5 (±0.3) km/h. Two photocells were used to measure and control the speed between trials (±10% of target speed).

For 3D motion analysis, anthropometric measurements (height, weight, leg length, and knee and ankle diameters) were taken for each subject, and 22 retro-reflective markers were placed bilaterally (on the shoe over the second metatarsal head and over the posterior calcaneus, lateral malleolus, lateral shank, lateral knee, lateral thigh, anterior superior iliac spine, posterior superior iliac spine, clavicula, sternum, seventh cervical vertebra, and tenth thoracic vertebra) on the subjects based on the Plug-in Gait full-body model (Vicon, Oxford, UK). An eight-camera system (Vicon T40, Vicon) and five force platforms (total length 5.7 m; AMTI, Watertown, MA, USA) were used to record the marker positions and ground reaction force data synchronously at 300 and 1500 Hz, respectively.

### Data analysis

Five clear force plate contacts of the right leg were selected for the analysis. Kinematic and kinetic analyses as well as a calculation of the position of the CoM were performed using the Vicon Nexus (v. 1.85, Oxford, UK). Marker trajectories and GRF data were low-pass-filtered using a fourth-order Butterworth filter with cut-off frequencies of 12 and 50 Hz, respectively. Foot contact and toe-off events based on the 20-N vertical force threshold level were used to calculate contact time, cadence and step length.

Leg compression in this study was determined directly from 3D motion analysis data as a distance vector between the proximal and distal end-points of the leg-spring^26,30^ rather than using a traditional spring-mass model^19,20^. The hip joint centre was used as a proximal end-point, but unlike earlier studies^26,30^ (with the direct 3D method) that used the centre of pressure location as a distal end-point, we selected the ankle joint centre for a distal end-point. This excludes any effect of possible shoe midsole deformation on leg compression values.

Leg stiffness (*k*_*leg*_) was then calculated by taking the ratio of the leg vector projected ground reaction force (GRF_proj_) to the compression of the leg at the instant when this compression was maximal^26^:

$${k}_{leg}=GR{F}_{proj}/le{g}_{comp}$$

We also report dimensionless leg stiffness (*k*_*leg_n*_) values to account for differences in the participants’ sizes^32^ (see supplementary material):

$${k}_{leg\_n}={k}_{leg}({L}_{leg}/BW)$$

where *L*_*leg*_ is the length of the leg during standing and *BW* is the body weight.

To determine the vertical impact load characteristics in the present study, the GRF data were exported from Vicon Nexus into Signal software (v.4.1, Cambridge Electronic Design, Cambridge, UK) for further analysis. The magnitude of the IP was defined as the first peak in the vertical ground reaction force curve (Fig. 1b). For two subjects whom we did not identify the IP for the MAX shoe condition, the IP was first determined for the CON shoe, and the time point of the IP was then used to determine the magnitude of the IP for the MAX shoe^33^. The average LR was defined as the rate of change of the vertical ground reaction force from 20% to 80% of the period from foot contact to IP^34^.

### Statistical analysis

Statistical tests were performed with IBM SPSS software (Version 22.0, Chicago, IL, USA). Shapiro-Wilk and Levene’s tests were used to confirm the normal distribution and the equality of variances, respectively. A two-way repeated-measures analysis of variance (ANOVA) was used to test the main and interaction effects of the two shoe types (CON and MAX) and the two running speeds (10 and 14.5 km/h). For significant events, the student’s paired *t*-tests were performed to determine the effect of shoe type at slower and faster running rates separately. *P* < 0.05 was considered significant. Symbols are used to describe statistically significant differences as follows: *P < 0.05; **P < 0.01, ***P < 0.001.

## Electronic supplementary material

Supplementary material

## Data Availability

The datasets generated and/or analysed during the current study are available from the corresponding author on reasonable request.
