# Supplementary material for: Running in highly cushioned shoes increases leg stiffness and amplifies impact loading
Source: Sci Rep. 2018 Nov 30;8:17496. doi: 10.1038/s41598-018-35980-6 (PMC6269547; doi:10.1038/s41598-018-35980-6)
Supplement: Supplementary file 1 — Supplementary material [file 41598_2018_35980_MOESM1_ESM.pdf]

Supplementary Materials for

Running in highly cushioned shoes increases leg stiffness and amplifies impact loading

Juha-Pekka Kulmala[1,2], Jukka Kosonen[3], Jussi Nurminen[1], Janne Avela[3]

[1] Motion Analysis Laboratory, Children's Hospital, University of Helsinki and Helsinki University Hospital, Helsinki, Finland.
[2] John A. Paulson School of Engineering and Applied Sciences, Harvard University, Cambridge, MA, USA.
[3] Neuromuscular Research Center, Biology and Physical Activity, Faculty of Sport and Health Sciences, University of Jyväskylä, Jyväskylä, Finland.

Corresponding author:

Dr. Juha-Pekka Kulmala
juha-pekka.kulmala@gmail.com

The PDF file includes:

- Figure S1. Mean data of the body's CoM (Centre of Mass) height **a** and displacement **b** trajectories for the CON and MAX shoes at slow and fast running speeds.

- Table S2. Dimensionless leg stiffness and leg compression data for the CON and MAX shoes at slow and fast running speeds.

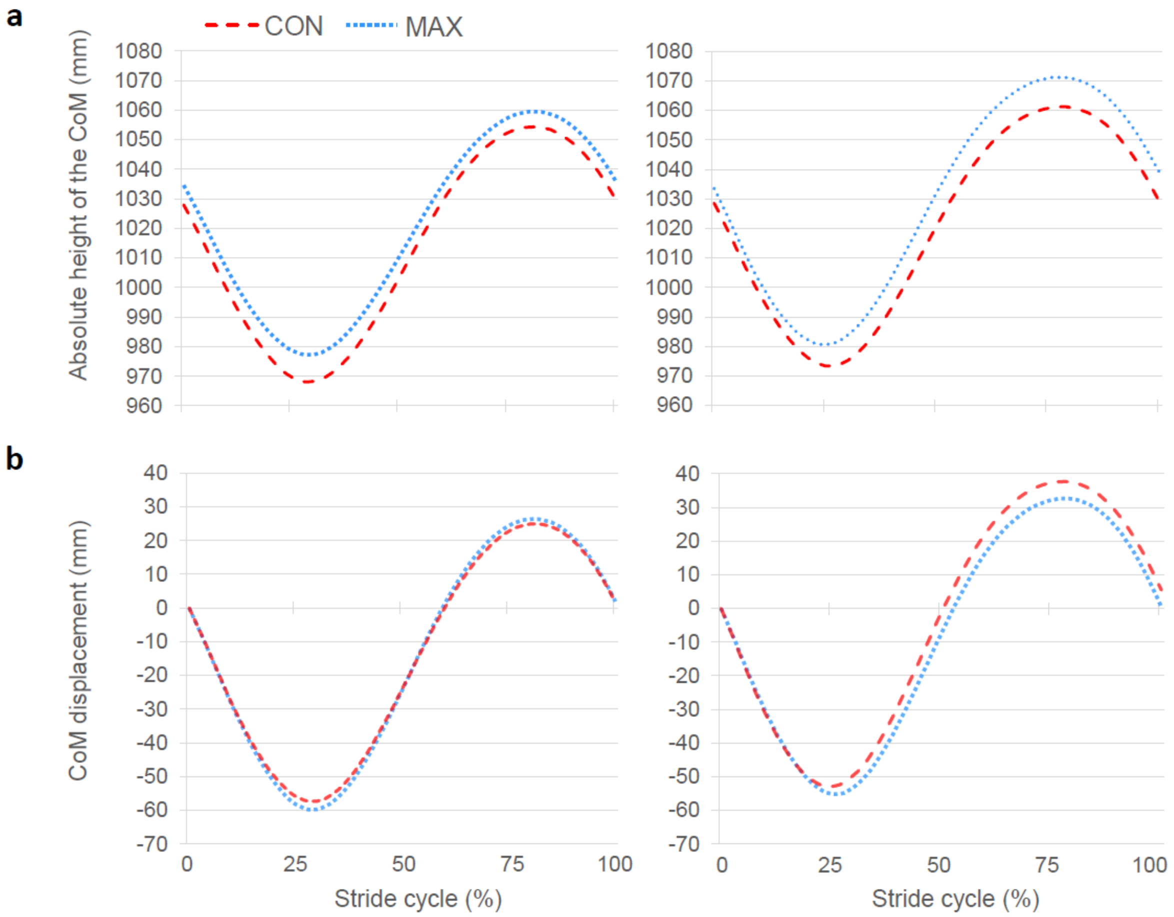

Figure S1. Mean data of the body's CoM (Centre of Mass) height **a** and displacement **b** trajectories for the CON and MAX shoes at slow and fast running speeds.

Table S1. Dimensionless leg stiffness and leg compression data for the CON and MAX shoes at slow and fast running speeds.

	**Slow speed (10 km/h)**			**Fast speed (14.5 km/h)**		
	**CON shoe**	**MAX shoe**	***t*-test**	**CON shoe**	**MAX shoe**	***t*-test**
Spring-like mechanics						
Dimensionless leg stiffness $^{++}$	26.0 (5.6)	26.7 (6.2)	0.208	28.5 (6.5)	30.1 (7.0)	0.006**
Dimensionless leg compression $^{++}$	0.100 (0.014)	0.096 (0.014)	0.029*	0.101 (0.015)	0.097 (0.016)	0.007**

Univariate difference between shoe conditions (*t*-test): $*p < 0.05$ and $**p < 0.01$.
The main effect for shoe conditions (a two-way repeated measures ANOVA): $^{++}p < 0.01$
Dimensionless leg stiffness was calculated by normalising leg stiffness (in kN/m) by body weight and leg length.
Dimensionless leg compression was calculated by normalising leg compression by leg length.
